# Effect of the COVID-19 Pandemic on Medical Student Performance and Evaluation Scores

**DOI:** 10.58624/SVOAMR.2024.02.011

**Published:** 2024-06-24

**Authors:** Erika R. Noel, Barry Mizuo, Loren G. Yamamoto, Kyle M. Ishikawa, John J. Chen, Kyra A. Len

**Affiliations:** 1 Department of Pediatrics, John A. Burns School of Medicine, University of Hawai`i, Honolulu, Hawaii, USA.; 2 Department of Pediatrics, John A. Burns School of Medicine, University of Hawai`i, Honolulu, Hawaii, USA.; 3 Loren G. Yamamoto, MD, MPH, MBA, Department of Pediatrics, John A. Burns School of Medicine, University of Hawai`i, Honolulu, Hawaii, USA.; 4 Department of Quantitative Health Sciences, John A. Burns School of Medicine, University of Hawai`i, Honolulu, Hawaii, USA.; 5 Department of Quantitative Health Sciences, John A. Burns School of Medicine, University of Hawai`i, Honolulu, Hawaii, USA.; 6 Department of Pediatrics, John A. Burns School of Medicine, University of Hawai`i, Honolulu, Hawaii, USA.

**Keywords:** SARS-CoV-2 Pandemic, COVID-19 Pandemic, Medical Education Curriculum Changes, USNBME, Third Year Clerkships, OSCE, USMLE Step 2 Examination, Patient Logs

## Abstract

In March 2020, the University of Hawaii John A. Burns School of Medicine suspended in person clinical teaching due to the SARS-CoV-2 (COVID) pandemic. During this period, virtual cases, telehealth participation, and online cases were incorporated into medical education. We have examined the effects of educational outcomes of third and fourth year students throughout clerkship performance, national standardized test scores, and our local fourth year OSCE examination. We found that USMLE step 2 scores were higher in the COVID-affected group. Patient logs in the COVID-affected group were lower for internal medicine, family medicine, OBGYN, and psychiatry clerkships. Clerkship performance grades in the COVID-affected group were lower for OBGYN and higher for surgery and psychiatry, but not different in other clerkships. USNBME subject specific examination scores in the COVID-affected group were higher for internal medicine, surgery, family medicine and psychiatry, but not different in all other specialties. For the fourth year OSCE, students in the COVID-affected group performed better on note taking and worse on physical examination. Future investigations will be needed to explore how our COVID-affected medical students perform in residency and beyond.

## Introduction

The SARS-CoV-2 (severe acute respiratory syndrome coronavirus 2) (also known as COVID-19 or abbreviated further as “COVID”) pandemic has affected medical education globally by limiting the opportunities for medical students to care for patients during their clinical rotations.^1–6^ On March 13, 2020, the U.S. Association of American Medical Colleges (AAMC) provided guidelines to consider suspending clinical rotations for medical students if there were difficulties placing students in healthcare settings.7

Given the shortage of personal protective equipment and limited COVID testing capabilities during this initial period, the medical students at the University of Hawaii John A. Burns School of Medicine (UHJABSOM) were suspended from their clinical rotations and transitioned to virtual learning on March 16, 2020. On March 17^th^, the AAMC recommended all medical schools suspend their clinical rotations.^7^

During this pandemic period, learning experiences shifted away from the clinical environment and clinical educators were forced to seek out alternative learning strategies including online clinical didactic sessions, the creation or utilization of available virtual cases, and increased involvement of medical students in telehealth medicine.^8–12^ UHJABSOM students continued virtual learning for 7 weeks until May 4, 2020 at which time the clinical rotation restrictions were lifted due to better availability of masks and personal protective equipment supplies. This new learning environment included faculty lectures given via video-conferencing, participation in telehealth, participation in virtual oral cases, and completion of online interactive cases depending on the clerkship rotation. This virtual curriculum was strikingly different from the traditional clinical clerkship environment where students learned by taking care of actual patients, working closely with their clinical preceptors, and immersing themselves in the team-based framework of the hospital.

To date, there are no studies that have provided a comprehensive overview of the effect of the COVID-19 pandemic on educational outcomes of students during their clinical years of training including comparisons of clerkship grades, U.S. National Board of Medical Examiner (USNBME) subject specific standardized student examination scores (also known as “shelf exams”), Objective Structured Clinical Skill exam (OSCE) performance (at the MS4 level), and U.S. Medical Licensing Examination (USMLE) step 2 clinical knowledge (CK) performance. The USMLE is a required exam for medical licensure in the U.S., which consists of three parts (steps). Step 2 is typically taken at the end of the 3^rd^ year or early in the 4^th^ year of medical school. Step 3 is taken just prior to or after the completion of the first year of post graduate residency training. The objective of this study is to provide a more global assessment of how the COVID-19 pandemic impacted the academic performance of medical students during their clerkship experience. Our assessment will include comparisons of USMLE step 2 CK scores, clerkship patient logs, USNBME subject specific standardized examination scores, clerkship grades, and OSCE scores amongst students at our institution who were removed from their in-person clinical experience at the onset of the pandemic and those who completed their clerkship experience prior to the pandemic. We hypothesized that students who were affected by the SARS-CoV-2 pandemic would not perform as well on their clerkships, the USMLE step 2 CK exam, and OSCE, compared to students who were able to have a traditional clerkship experience unaffected by the SARS-CoV-2 pandemic.

## Methods

The University of Hawaii John A. Burns School of Medicine (UHJABSOM) is a state-funded medical school where the majority of the students are residents of the state of Hawaii (50^th^ state in the U.S.). The students spend the first two years with a problem based learning (PBL) curriculum and the third and fourth years in their clinical years are spent in the clinics and hospitals. We performed a retrospective review of 3rd year medical student (MS3) exam scores and clinical evaluations in the academic years 2017–2018, 2018–2019 and 2019–2020, with students affected by the pandemic with online curriculum being our study group. We collected data on student clerkship grades, clerkship patient logs, USNBME subject specific examination student scores, USMLE step 2 CK scores, and MS4 OSCE grades. For the MS4 year OSCE mean scores from the components of history taking, communication and interpersonal skills (CIS), physical examination, and note taking scores were compiled.

At UHJABSOM, a modified clerkship schedule was instituted with the primary goal of mitigating the detrimental effects of the SARS-CoV-2 (COVID) pandemic on student experiential learning while on their clinical rotations. Their rotation schedule was adjusted so that they all had a portion of their remaining clerkships for their MS3 year transitioned to virtual so that each student would miss only 1 to 3 weeks of an inpatient experience, but not an entire clerkship rotation. For example, a student would do 2 weeks of virtual pediatrics, internal medicine, and then surgery, and when we returned to do in-person clinical rotations, they did 2 weeks of inpatient pediatrics, internal medicine, and surgery, respectively. Students who were scheduled for their outpatient semester in the spring may have missed 7 weeks of their outpatient rotation, but the rest of the semester was spent in person.

Students who had modified rotations either in the inpatient or outpatient rotation during this 7 week period were identified as the COVID-affected group, and students who were able to complete their entire rotation in person were identified as the COVID-unaffected group. This specifically applies only to the 2019–2020 MS3 class. The MS3 rotations and instruction for the 2017–2018 and 2018–2019 MS3 classes were not affected by COVID-19.

Within each rotation, the scores of students who were affected by COVID were compared to those who were unaffected by COVID. When scores were not significantly different among years, student scores from 2017–2018 and 2018–2019 were combined with those who were not affected by COVID in 2019–2020 to form the comparison group.

To compare scores between COVID-affected and -unaffected students, Chi-square (or Fisher’s exact test when applicable) was used for categorical variables (e.g., clerkship grades) and T-tests were used for continuous variables (e.g., patient logs and USNBME subject specific student scores). USMLE step 2 CK scores and OSCE scores were compared by year, considering all those in 2019–2020 to be COVID-affected. A two-sided p-value of less than 0.05 was regarded as statistically significant.

This study was approved by the University of Hawai`i Institutional Review Board as an exempt study (protocol number 2021–00194).

## Results

There were a total of 71 students in the class of 2017–2018, 68 students in the class of 2018–2019 and 75 students in the class of 2019–2020. In the COVID-affected group, there were 39, 55, 43, 33, 39, and 47 students who had COVID-modified clinical rotations in pediatrics, internal medicine, surgery, family medicine, obstetrics/gynecology, and psychiatry, respectively. For some students, data was not available for all categories, so they were not included in the data tabulations. Note that since the period of COVID clinical rotation restrictions was March 16, 2020 to May 4, 2020, a student could have had a COVID-modified (restricted) pediatric rotation, while having a conventional (unmodified/ unrestricted) surgery rotation (as an example).

**Figure 1.**
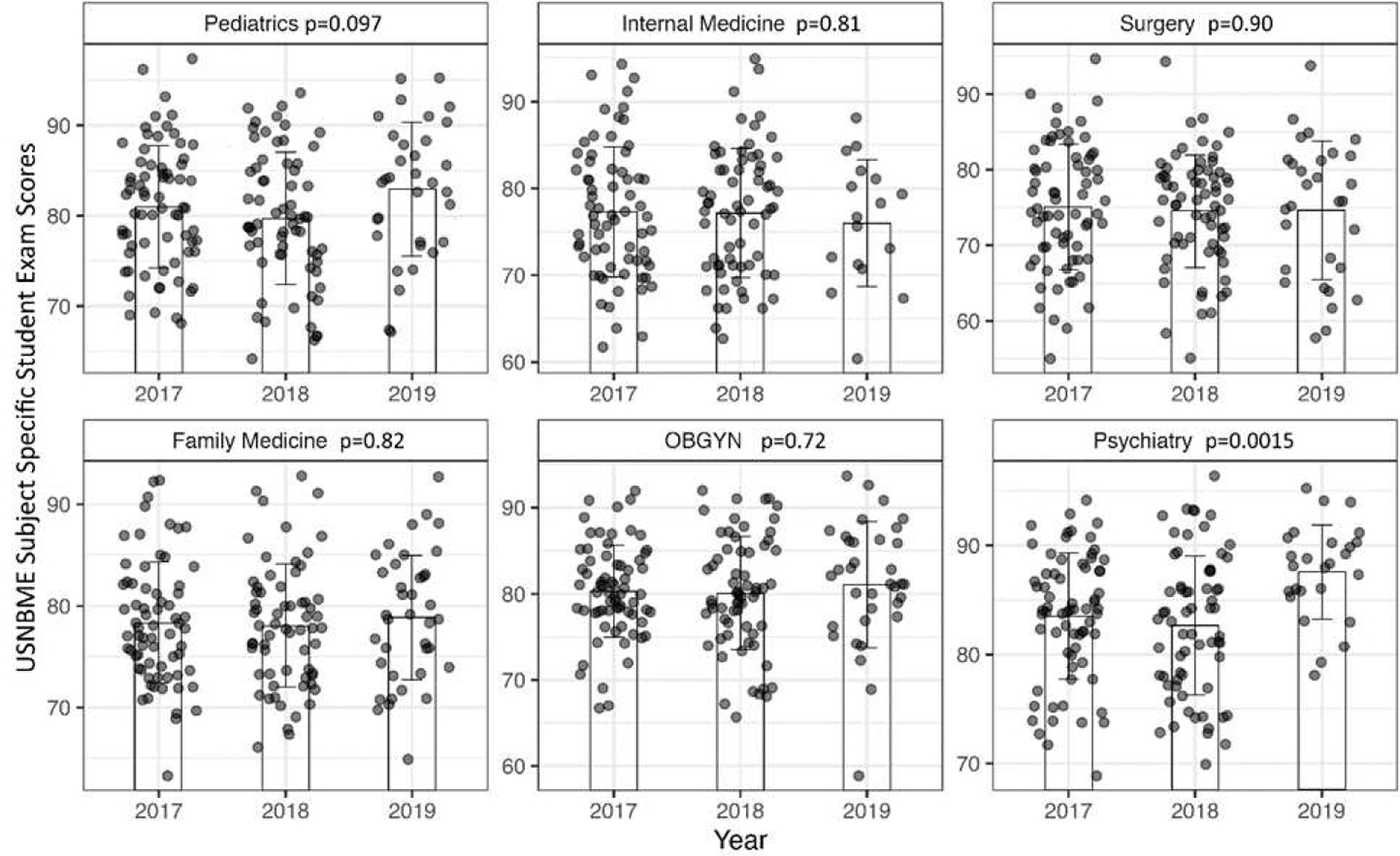
Evaluation of Homogeneity of USNBME Subject Specific Student Exam Scores Among Years. For the 2019 group, the plot excludes students who had COVID-affected (modified/restricted) clinical rotations for the respective specialties. The bars represent the mean. The error bars represent the standard deviation. The p values are derived from analysis of variance (ANOVA). Psychiatry had significantly higher scores in the 2019 COVID year compared to the pre-COVID years. The scores in the other specialties were not significantly different. Kruskal-Walis shows similar results. The dots are data points which are randomly offset horizontally to avoid overlapping. The years 2017, 2018, and 2019 refer to the academic years 2017–2018, 2018–2019, 2019–2020, respectively for the MS3 classes.

To test the homogeneity of USNBME subject specific exam scores among years, Figure 1 graphs the spread of the individual scores, the means, and standard deviations for each of the clinical specialty subjects by year. Analysis of variance (ANOVA) shows that psychiatry was the only clinical specialty that showed a significant difference over the years with the highest score in 2019–2020.

**Figure 2.**
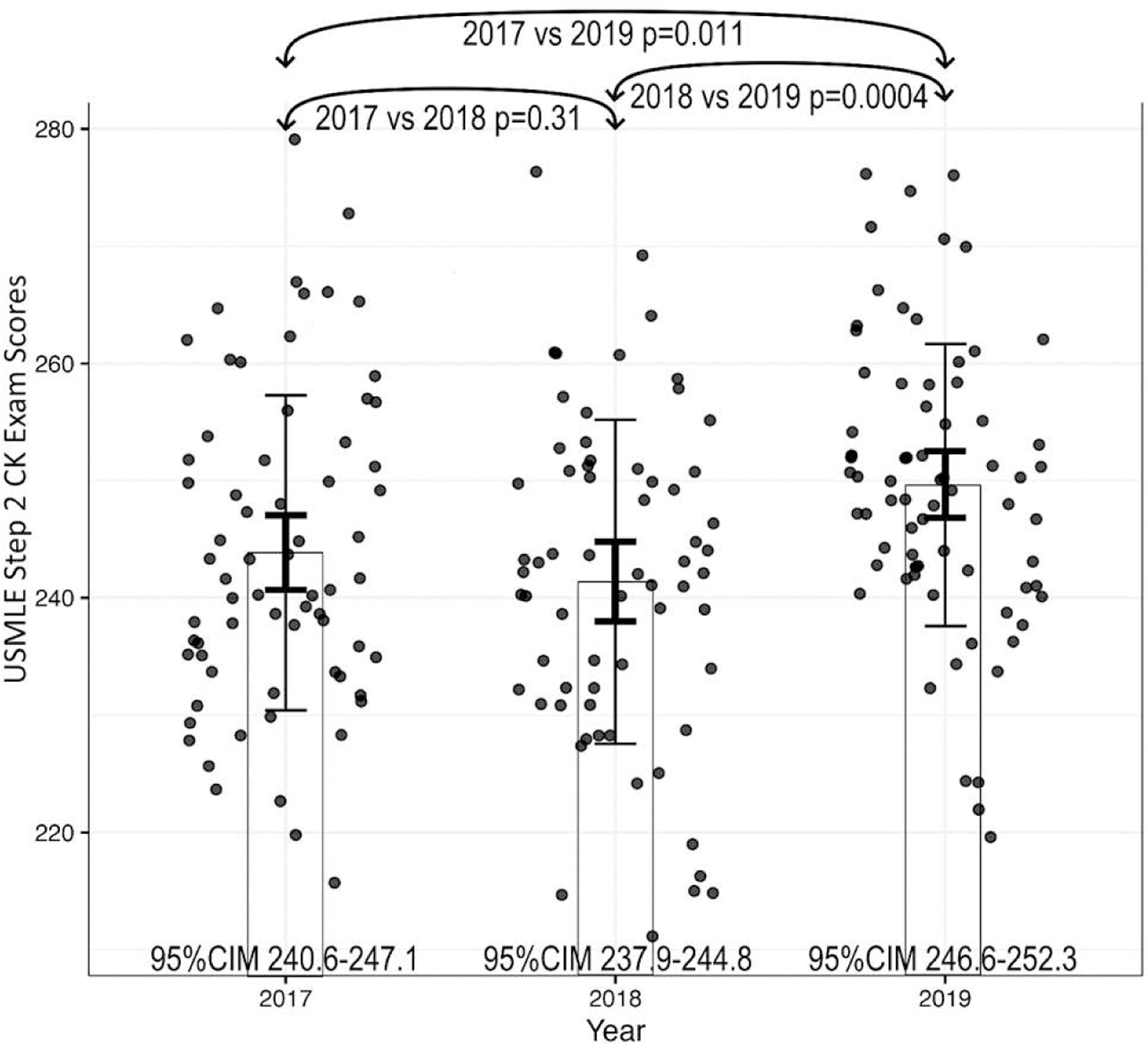
USMLE Step 2 CK Exam Scores Among Years. The bars show the group means. By analysis of variance (ANOVA), p=0.0014. The thin error bars represent the standard deviation. The thick error bars represent the 95% confidence interval of the mean (95%CIM). The dots are data points which are randomly offset horizontally to avoid overlapping. The ANOVA and the T-test between years results indicate that the 2019 year has a significantly higher score than both the 2017 and 2018 years. The years 2017, 2018, and 2019 refer to the academic years 2017–2018, 2018–2019, 2019–2020, respectively for the MS3 classes. Note that only the 2019–2020 class contains students who have had a combination of some COVID-affected rotations (restricted/modified) and COVID-nonaffected rotations. The 2017–2018 and 2018–2019 classes were not affected by COVID in their MS3 years.

Clerkship patient logs, clerkship grades, and USNBME subject specific scores of students are summarized in Table 1. Patient logs in the COVID-affected group were lower for internal medicine, family medicine, OBGYN, and psychiatry, but not different for pediatrics and surgery. USNBME subject specific scores of students in the COVID-affected group were higher for internal medicine, surgery, family medicine, and psychiatry, but not different in all other specialties. Clerkship performance grades in the COVID-affected group were lower for OBGYN and higher for surgery and psychiatry, but not different in other clerkships. USMLE step 2 scores were higher in the COVID-affected group, as seen in Figure 2.

The MS4 OSCE results can be seen in Table 2. Students in the 2019–2020 COVID-affected class performed better on note taking and worse on physical examination. History taking had a significant p value and the highest score was achieved during the 2019–2020 year; however, the 2018–2019 score was low suggesting that the most appropriate conclusion regarding this p-value is that the 2018–2019 score was significantly lower than the other two years.

## Discussion

Our study provides a comprehensive overview of the COVID-19 pandemic’s impact on medical student educational outcomes during their clinical training. Others have tried to assess the impact of the COVID-19 pandemic on medical student education by focusing on issues such as student perception of how the pandemic impacted their learning, mental health concerns, and their specialty choice.^13–19^ Few studies have evaluated the impact the pandemic has had on academic performance during the clinical training years of medical school. For example, Coronel-Couto et al., did not find a significant difference in how well their institution’s students scored on the USNBME subject specific exam when the internal medicine clerkship experience was shortened due to the pandemic compared to students the following year who had a longer and more typical clinical experience.^20^ Studies such as this specifically focused on individual clerkship outcomes, but failed to look at whether these outcomes were perpetuated amongst other clinical rotations that students are expected to complete as part of their clinical training.

We hypothesized that students who were removed from clinical rotations at the inception of the COVID-19 pandemic would not perform as well on their exam scores and other graded aspects of their clerkship experience. Contrary to our hypothesis, we often saw the opposite outcome. COVID-affected students achieved a higher internal medicine, surgery, family medicine, and psychiatry USNBME subject specific exam scores. There was no significant change in their pediatric, or obstetrics/gynecology subject specific scores compared to the unaffected cohort. The higher psychiatry USNBME subject specific exam score in the COVID-affected group, could be due to a class effect, rather than a COVID effect because Figure 1 demonstrated that the 2019–2020 class had a significantly higher psychiatry USNBME subject specific exam score even after removing the students who had COVID-affected psychiatry clerkships.

Our results contradict what Hanson et al., reported in which students who had a predominantly virtual pediatric clerkship experience during the start of the pandemic were more likely to fail their USNBME subject specific examination.^21^ This unexpected outcome may be attributed to changes that UHJABSOM made in response to the COVID-19 pandemic. A modified schedule was instituted to minimize the missed in-person time on each clerkship. Each student missed no more than 3 weeks on a single clerkship despite being removed from clinical rotations for 7 weeks. Additionally, online activities were made available to the students to supplement their education since they were restricted from being present in the clinical environment. These activities included virtual lectures, online interactive web-based cases, and virtual one on one meetings with faculty preceptors. The modified schedule and supplemental learning activities might be one reason why our students whose schedule was impacted by the COVID-19 pandemic were still able to acquire the medical knowledge to perform the same or better on the various USNBME subject specific exams compared to students who completed the traditional clerkship experience. This suggests that clinical restrictions can be overcome by more robust alternatives to actual clinical patient care.

When analyzing overall clerkship grades, there was only a significant difference identified in three clerkships. The surgery clerkship grade in the COVID-affected cohort was higher than the unaffected group. Significantly higher USNBME surgery shelf exam scores that were seen in the COVID-affected group may have contributed to the higher overall grades since 30% of the surgery clerkship grade is based on the USNBME subject specific exam score. Our findings are consistent with what Prigoff, et al., discovered in that a higher percentage of students who experienced a COVID-affected interruption to their surgery clerkship experience achieved honors as compared to a cohort of students who rotated through the surgery clerkship prior to the start of the pandemic.^22^ Psychiatry grades were also higher which could be due to the higher overall USNBME subject specific exam score in Psychiatry for the 2019–2020 class even after the psychiatry COVID-affected students were removed suggesting that this could be a class effect, rather than a COVID-effect. For OBGYN, the clerkship grades were lower in the COVID-affected cohort which might have been the result of less clinical exposure in this particular clerkship without any major adjustments made in how students were graded. Apart from the OBGYN clerkship, COVID-affected students in our study did not have a lower clerkship grade even with less clinical exposure. This partially may have been related to the fact that four out of six clerkship rotations had higher USNBME shelf exam scores. Depending on the clerkship specialty, the shelf examination constitutes as much as 20% to 30% of the final clerkship grade. Although the COVID-affected students performed higher on the USNBME subject specific exams, the decrease in clinical exposure still may have resulted in less favorable clinical performance evaluations for internal medicine and family medicine resulting in an overall similar grade to the unaffected cohort.

The USMLE Step 2 CK exam is a clinical exam (covering all clinical areas) that our students take at the end of their 3rd year after completing all required clerkships. This exam is often thought to be a more clinically focused exam. Despite being affected by less clinical exposure, 2019–2020 medical students achieved significantly higher USMLE step 2 CK scores compared to prior students not affected by the pandemic. Though it is difficult to determine which variable influenced this positive trend the most, it is possible that the increased study time that the pandemic afforded students may have been more helpful than the practice of caring for patients in-person. Prior studies have shown the optimal time to take the USMLE Step 2 CK exam is early during their senior year and that less than two weeks of preparation seemed to be better than more than 2 weeks of study time.^23–24^ Though our COVID-affected students had considerably more than a 2 week study time period, they also had to focus their studying efforts on passing the USNBME subject specific shelf exams and completing online curriculum requirements. Nevertheless, the 2019–2020 students achieved significantly higher USMLE step 2 scores compared to 2017–2018 and 2018–2019 students who completed the USMLE step 2 exam without the same interruption in their clinical experience.

Students take their OSCE exam at the start of their fourth year. COVID affected students performed worse on the physical examination portion of this exam. This was likely a result of students having less time to practice their clinical skills on actual patients. It was surprising though that the COVID affected students still had a higher note taking score on the OSCE. One hypothesis for this finding is that feedback on clinical rotations resulted in students writing more succinct notes which may be looked upon less favorably on a standardized OSCE exam. This is in contrast to Tzeng, et al, who observed a decrease in national OSCE scores in their medical institution in Taiwan during the pandemic.^25^ The USMLE Step 2 CS (clinical skills) exam was the primary method for U.S. medical schools to determine student competency in clinical skills in a more objective, standardized fashion. However, since the USMLE Step 2 CS exam was canceled in January 2021, there is a greater responsibility on medical schools to assess the clinical skill competency of their students.^26^ Students who experienced reduced clinical exposure due to pandemic restrictions might need additional clinical skills practice to ensure their physical examination skills are adequate before they start residency.

Students in the COVID affected group saw fewer patients in the clinical clerkship rotations of internal medicine, family medicine, obstetrics/gynecology, and psychiatry, with no significant difference in pediatrics and surgery. Modifications to the medical school curriculum were observed globally during the pandemic with changes focused on restricting the amount of in-person clinical care that students were accustomed to. Therefore, it was not surprising to see that our students saw fewer patients in comparison to previous years or even their own classmates whose clerkship experience was not affected by the pandemic. Limitations of this part of the study include the accuracy of patient logs since it is entirely dependent on individual students to be responsible for logging all of their patient encounters correctly (rather than the bare minimum that was required). Variability in how students logged their patient encounters likely led to inaccuracies in the total number of patients that were recorded. We anticipated seeing a decrease in patient logs across all clerkships. However, there was no decrease seen in the pediatrics clerkship. Lack of a significant decrease in their patient logs during the pandemic could be attributed to the instructions given on these clerkships to log required clinical conditions encountered rather than every patient that is seen.

The COVID-19 pandemic has forced undergraduate medical education to evolve rapidly, leading to unforeseen consequences in how medical students acquire essential clinical knowledge and skills. This study provides a comprehensive overview of how the pandemic may have impacted student learning based on differences seen in various clinical assessments during the MS3 and MS4 academic years at our institution.

## Conclusion

In conclusion, despite having less clinical exposure, COVID-affected students had higher clerkship grades in surgery and psychiatry, and higher standardized test scores including the USMLE Step 2 scores, 4 out of 6 of the USNBME subject specific exam scores, and the note taking score on the MS4 OSCE in comparison to the cohort of students unaffected by COVID; with just a few negative outcomes. Future investigations exploring how our COVID-affected medical students perform clinically in residency and beyond may help identify other ramifications the pandemic had on their medical training.

## Figures and Tables

**Table 1.** Comparisons of Student Performance Measures Between COVID-Affected vs -Unaffected Students [n]. Patient log refers to the number of patient encounters logged by students for the respective specialty rotation. The USNBME score is for the respective subject specific exam.

	Unaffected^1,2^	COVID-Affected^1,2^	p-value^3^
**Pediatrics**			
Patient Log	68 ± 44 [169]	76 ± 35 [39]	0.22
USNBME score	81 ± 7 [169]	82 ± 6 [39]	0.20
Clerkship Grade^4^			0.65
= Pass	115/169 (68%)	28/39 (72%)	
= Honors	54/169 (32%)	11/39 (28%)	
**Internal Medicine**			
Patient Log	213 ± 113 [147]	167 ± 81 [52]	0.002
USNBME score	77 ± 7 [151]	81 ± 7 [55]	0.0006
Clerkship Grade			0.35
=Pass	96/151 (64%)	29/55 (53%)	
=High Pass	3/151 (2%)	1/ 55 (2%)	
=Honors	52/151 (34%)	25/55 (45%)	
**Surgery**			
Patient Log	59 ± 34 [165]	61 ± 33 [43]	0.80
USNBME score	75 ± 8 [165]	80 ± 7 [43]	0.0005
Clerkship Grade			0.020
=Pass	72/165 (44%)	9/43 (21%)	
=High Pass	44/165 (27%)	14/43 (33%)	
=Honors	49/165 (30%)	20/43 (47%)	
**Family Medicine**			
Patient Log	270 ± 107 [169]	131 ± 74 [29]	<0.0001
USNBME score	78 ± 6 [175]	81 ± 5 [33]	0.013
Clerkship Grade			0.26
=Pass	119/175 (68%)	18/33 (55%)	
=High Pass	11/175 (6%)	4/33 (12%)	
=Honors	45/175 (26%)	11/33 (33%)	
**Obstetrics/Gynecology**			
Patient Log	181 ± 98 [168]	137 ± 63 [35]	0.001
USNBME score	80 ± 6 [170]	82 ± 5 [39]	0.17
Clerkship Grade			0.0027
=Pass	9/170 (5%)	8/39 (21%)	
=High Pass	91/170 (54%)	22/39 (56%)	
=Honors	70/170 (41%)	9/39 (23%)	
**Psychiatry**			
Patient Log	102 ± 55 [144]	74 ± 37 [42]	0.0002
USNBME score	84 ± 6 [161]	87 ± 4 [47]	0.0002
Clerkship Grade^4^			0.0008
=Pass	67/161 (42%)	7/47 (15%)	
=Honors	95/161 (58%)	40/47 (85%)	

1 Mean ± SD for continuous scores.

2 n / N (%) for categorical scores.

3 T-test, Chi-square (or Fisher’s test as appropriate).

4 Clerkship grades for pediatrics and psychiatry were limited to Pass and Honors (i.e., no “High Pass” grades were on the student records).

**Table 2.** OSCE Scores: Means ± standard deviations and p values by analysis of variance (ANOVA) for the years 2017–2018, 2018–2019, and 2019–2020 [n]. Note that the 2019–2020 class contains students who have had some COVID-affected rotations (restricted/modified).

OSCE Score Category	2017–2018 [71]	2018–2019 [66]	2019–2020 [71]	p-value
History Taking	71.8 ± 16.7	64.3 ± 10.7	73.6 ± 8.0	<0.0001
Physical Exam	74.6 ± 18.0	73.0 ± 12.4	62.6 ± 12.3	<0.0001<0.0001
Communication and Interpersonal Skills	88.9 ± 19.4	84.8 ± 12.1	85.1 ± 6.5	0.14
Note Taking	80.9 ± 17.7	83.6 ± 11.3	89.8 ± 5.3	<0.0001
